# Subjective health complaints in older adolescents are related to perceived stress, anxiety and gender – a cross-sectional school study in Northern Sweden

**DOI:** 10.1186/1471-2458-12-993

**Published:** 2012-11-16

**Authors:** Maria Wiklund, Eva-Britt Malmgren-Olsson, Ann Öhman, Erik Bergström, Anncristine Fjellman-Wiklund

**Affiliations:** 1 Umeå Center for Gender Studies, Umeå University, Umeå, Sweden; 2 Department of Community Medicine and Rehabilitation, Physiotherapy, Umeå University, Umeå, Sweden; 3 Department of Public Health and Clinical Medicine, Epidemiology and Global Health, Umeå University, Umeå, Sweden; 4 Department of Clinical Sciences, Pediatrics, Umeå University, Umeå, Sweden

**Keywords:** Sweden, Adolescent, School students, Self-reported health, Psychosomatic, Stress, Pain, Mental health, Anxiety, Depression

## Abstract

**Background:**

Negative trends in adolescent mental and subjective health are a challenge to public health work in Sweden and worldwide. Self-reported mental and subjective health complaints such as pain, sleeping problems, anxiety, and various stress-related problems seem to have increased over time among older adolescents, especially girls. The aim of this study has therefore been to investigate perceived stress, mental and subjective health complaints among older adolescents in Northern Sweden.

**Methods:**

Data were derived from a cross-sectional school-based survey with a sample consisting of 16–18 year olds (n = 1027), boys and girls, in the first two years of upper secondary school, from different vocational and academic programmes in three public upper secondary schools in a university town in northern Sweden. Prevalence of perceived stress, subjective health complaints, general self-rated health, anxiety, and depression were measured using a questionnaire, including the Hospital Anxiety and Depression Scale (HADS).

**Results:**

A large proportion of both girls and boys reported health complaints and perceived stress. There was a clear gender difference: two to three times as many girls as boys reported subjective health complaints, such as headache, tiredness and sleeping difficulties and musculoskeletal pain, as well as sadness and anxiety. High pressure and demands from school were experienced by 63.6% of girls and 38.5% of boys. Perceived stress in the form of pressure and demands correlated strongly with reported health complaints (r = 0.71) and anxiety (r = 0.71).

**Conclusions:**

The results indicate that mental and subjective health complaints are prevalent during adolescence, especially in girls, and furthermore, that perceived stress and demands may be important explanatory factors. Future studies should pay attention to the balance between gender-related demands, perceived control and social support, particularly in the school environment, in order to prevent negative strain and stress-related ill-health. The gender gap in subjective adolescent health needs to be further explored.

## Background

Adolescent mental and subjective health has become a public health concern in Sweden and worldwide [1-4]. In Sweden, self-reported mental and subjective health complaints, such as pain, sleeping problems, anxiety, and various stress-related problems are common and seem to have increased over time among older adolescents, especially girls [5-9]. Similar trends are found across the Nordic countries, the rest of Europe, and North America indicating the need to address socio-contextual factors in the study of gender health inequalities during adolescence [10-13]. However, time trends are found to be discrepant and subject to change [5,6]. For example, a study from 2004 observed a rise of conduct- and emotional problems in UK adolescents over a 25-years period [14]; whereas more recent ratings indicated that children’s mental health problems now may have “peaked” [15]. In comparison, a national Dutch study shows divergent trends in adolescent mental health in terms of sex/gender with a decline in boys’ externalization problems, but an increase in girls’ internalization problems [16].

In Sweden, adolescents’ “stress” and mental health are now on the public health agenda [7,17]. Repeated Internet-based surveys found that perceived stress and psychosomatic health problems peak in adolescent girls at 16–18 years of age [8]. In this age group 37% of the girls and 22% of the boys considered themselves as being “very often” stressed. Also the longitudinal national *Survey of living conditions among children* shows high prevalence of self-reported stress in female upper secondary school students [18]. In a sample of 16-year old upper secondary school students in Stockholm, one-third reported serious stress-symptoms with 14.2% of the girls and 2.6% of the boys exceeding the set cut-off for chronic stress [19]. Perceptions of high demands, low global self-esteem, sleep disturbances and poor social support seemed to predict their stress symptoms.

In line with these empirical findings, adolescence in industrialized countries is commonly described as an important period of development characterized by tumultuous transition and “storm and stress” – although such notions also are questioned [20-22]. During adolescence, health and resilience develop in a manner closely linked to the social context such as family, peers, partners and school. However, this period can also be a time of psychological distress [19,23-27]. Adolescence is viewed as a critical period for early onset of mental illness [28]. Despite being heterogeneous as a group, adolescents share rapid physical, cognitive, emotional and social changes [21,29]. Adolescents’ health related quality of life is found to be influenced by development of self-governance and autonomy, position in peer-group, intimacy and sexuality as well as by physical maturation and a changing body [29]. Late adolescence can be a lonely time in life, with what is often a concomitant increase in various stressors and demands [21,29,30]. Adolescent stress appears to be linked to multiple stressors and areas such as school performance, partner relationships, bullying and peer pressure, financial problems, emerging adult responsibilities and worries about the future [21,31]. When placing these aspects in a wider social context, young people’s lives and challenges seem even more complex [31,32]. Social aspects of gender are also seen as interlinked with experienced stress and health-problems [31,33,34]. “Gendered pathways in school burnout” is a recently raised topic in research on adolescent stress [13].

Co-occurrence of physical and psychological health problems is common among adolescents [35] and associations between various stressors, perceived stress and subjective health complaints have been observed [24,36]. Frequent subjective health problems are headache, abdominal pain, musculoskeletal symptoms, sleeping difficulties and nervousness [37]. Multiple pain problems, as an example, have negative psychosocial consequences and impact on quality of life [38]. In addition, pain problems often persist into early adulthood [39]. Adolescent sleep disturbances are also found to predict sleeping disturbances later in life [40,41]. Moreover, longitudinal studies indicate that mild psychiatric symptoms early in life such as anxiety, insomnia or sub-threshold depression increase the risk of psychiatric conditions later in life [42-44].

These negative trends in adolescent health development are a challenge to public health work, as well as to society at large [2,9]. Explanatory factors regarding decreased subjective wellbeing and health complaints in young people have largely yet to be explored, especially the explanation behind the striking gender differences [9,10]. A number of epidemiological studies have been conducted among Swedish children and adolescents in primary and lower secondary schools, including some in northern Sweden [5,26,45-47], but studies among older adolescents are still scarce in the Umeå region. The aim of this study is to investigate the prevalence of perceived stress, mental and subjective health complaints – including possible differences between boys and girls – among upper secondary school adolescents in northern Sweden.

## Methods

### Study setting and procedure

This cross-sectional school study is part of a larger research project, Stress and Health among Youth in Northern Sweden (Umeå SHY), the purpose of which is to study stress, body and health among young people; and to develop and evaluate health-promotion intervention models within youth- and school health services. The project is a collaboration initiative between the university, the county council and the municipality in the region. The setting is the municipality of Umeå, a university city in northern Sweden with about 100 000 inhabitants. In Umeå there are both public and private upper secondary schools. At the time of the study 76% of students in the municipality attended public upper secondary schools, with 53% girls and 47% boys (School Statistics).

In 2007–2008, a classroom survey was conducted at three of the major public schools in Umeå municipality. The reason for this sample was to establish a baseline reference for a stress-management intervention conducted at the same schools. Each school offered a range of academic and vocational secondary school programmes. Vocational programmes were, and still are, directed towards work in areas such as the building-, service-, or health sectors which, not demand higher education. In contrast, academic programmes aim to prepare students for higher academic education in fields such as economics, media, the arts and the natural or social sciences.

The study was conducted in close cooperation with the school health services. Prior to the survey, the main heads of school were contacted, and granted permission for the classroom survey. All primary teachers at each educational programme and class were informed and invited to participate with their respective class. Teachers who expressed an interest were then contacted and arranged time for the survey. Before participation, students received written and oral information about the study, including information about confidentiality and the right not to participate. Information was also available to adolescents and parents on the schools’ Internet homepages. The survey was supervised by a member of the research team who also answered questions from the students. The questionnaire took about 30 minutes to complete, and was finished during a school lesson. The questionnaire was anonymous and no records or codes were obtained. The study was approved by the Regional Ethical Review Board in Umeå, Sweden (Dnr 05-045 M).

### Sampling and participants

There were a total of 2721 students in the first two years of upper secondary school at the three invited schools: 53% girls and 47% boys; 69% in academic programmes and 31% in vocational programmes. For practical reasons, 1545 of the students were not available to participate in the survey because of absence due to internship outside of school (vocational programmes), or because their teachers did not answer our invitation or had no possibility of allocating time for the classroom survey. Questionnaires were thus administered to 1176 students across 49 classes. In total, 1033 of these students answered the questionnaire, which resulted in a response rate of 87.8% in the participating classes. Non-participation was due to absence from school on the day the questionnaire was administered (n = 132). Answers from six students were excluded because they fell outside the selected sample of 16–18-year-olds: 15 years (n = 2), 19 years (n = 2) and 20 years (n = 2). Five questionnaires were excluded due to missing data. In total, 1027 questionnaires were included in the analysis.

### Measures

The questionnaire consisted of both established instruments and questions formulated specifically for this study. Questions covered socio-demographics, subjective health complaints, perceived stress [48], sleep, general self-rated health, medication, anxiety and depression [49,50]. The questionnaire comprises more areas than analyzed in the present study.

### Socio-demographics

Socio-demographics were described by sex, age, educational programme, family situation and country of birth.

### Subjective health complaints

Subjective health complaints was measured with a 16-item symptom checklist consisting of musculoskeletal pain in different parts of the body, headache, stomach and heart symptoms, symptoms of upper respiratory infection, tiredness/fatigue, nausea, dizziness and sadness. Six-month symptom prevalence was estimated through the question: “In the past six months, have you had the following problem?” Response alternatives were “never”, “seldom”, “sometimes”, “fairly often” and “very often”. For data analysis (Chi^2^ tests) the response alternatives were trichotomized as “never/seldom”, “sometimes” and “fairly often/very often”.

The factor analysis on subjective health complaints yielded three factors with eigenvalues greater than one (Table 1). The first factor was termed “psychosomatic symptoms” and consisted of five items (eigenvalue 6.07, Cronbach’s alpha 0.77). The second factor was named “headache/upper respiratory infection symptoms” and consisted of four items (eigenvalue 1.33, Cronbach’s alpha 0.75). The third factor was termed “musculoskeletal pain symptoms” and consisted of four items (eigenvalue 1.16, Cronbach’s alpha 0.76) (Table 1). Selected factors with an eigenvalue greater than 1 explained 53.5% of the variance. Three items (dizziness, stomach pain and sleeping problems) did not reach factor loading > 0.5 and were therefore excluded.

**Table 1:** Factor analysis using a rotated component matrix for subjective health complaint variables (factor loading >0.50 is cut-off point for inclusion)

**Subjective health complaint variables**	**Factor loading**		
	**Factor 1**	**Factor 2**	**Factor 3**
***Psychosomatic symptoms***	Eigenvalue 6.07 Cronbach’s alpha 0.77		
More tired than before	0.560		
Sadness	0.659		
Acid stomach, stomach ache	0.667		
Pain and aches in heart and chest	0.692		
Palpitations and extra heart beats	0.678		
***Headache/upper respiratory infection symptoms***		Eigenvalue 1.33 Cronbach’s alpha 0.75	
Headache		0.526	
Cold symptoms		0.799	
Cough and hoarseness		0.793	
Nausea		0.539	
***Musculoskeletal pain symptoms***			Eigenvalue 1.16 Cronbach’s alpha 0.76
Neck and shoulder pain			0.616
Low back pain			0.690
Pain in extremity joints			0.774
Muscle pain			0.754

Selected factors with an eigenvalue greater than 1 explained 53.5% of the variance.

### Perceived stress including sleep

Perceived stress was measured using a stress instrument developed by Lindblad et al. [48]. The instrument includes 16 items: 12 items on perceived stress and four items on sleep. Recall time was the previous weeks. Response alternatives were “never”, “seldom”, “sometimes”, “often”, and “always”. For data analysis (Chi^2^ tests), response alternatives were trichotomized as “never/seldom”, “sometimes” or “often/always”.

The factor analysis on perceived stress and sleep yielded three factors (Table 2). The first factor, “pressure and demands” included seven items (eigenvalue 5.97, Cronbach’s alpha 0.83). The second factor, “activation/high tempo” included five items (eigenvalue 1.41, Cronbach’s alpha 0.72). The third factor, “sleep problems” included four items (eigenvalue 1.04, Cronbach’s alpha 0.82). Selected factors with an eigenvalue greater than 1 explained 52.7% of the variance.

**Table 2:** Factor analysis using a rotated component matrix for perceived stress variables (factor loading >0.50 is cut-off point for inclusion)

**Perceived stress variables**	**Factor loading**		
	**Factor 1**	**Factor 2**	**Factor 3**
***Pressure and demands***	Eigenvalue 5.97 Cronbach’s alpha 0.83		
I don’t have enough time	0.754		
I feel under pressure from school demands	0.698		
I feel helpless	0.671		
I never feel really free	0.641		
I feel under pressure from demands at home	0.563		
I feel under pressure from my inner demands	0.536		
I don’t feel rested after sleep	0.531		
***Activation/high tempo***		Eigenvalue 1.41 Cronbach’s alpha 0.72	
I rush even if I don’t have to		0.721	
I keep a high speed all the day		0.700	
I eat rapidly even if I don’t have to		0.576	
I do many things at the same time		0.551	
I find it difficult to relax		0.533	
***Sleep problems***			Eigenvalue 1.04 Cronbach’s alpha 0.82
I sleep restlessly and shallow			0.763
I have difficulty falling asleep			0.739
I wake up early in the morning			0.606
I feel restless			0.524

Selected factors with an eigenvalue greater than 1 explained 52.7% of the variance.

### General self-rated health

General self-rated health was measured using one statement “I perceive my health as” with four response alternatives: “very good”, “fairly good”, “not quite good” and “not good at all”. The response alternatives were dichotomized into good (“very good/fairly good”) and bad (“not quite good/not good”) health. The question about self-reported health is similar to the question used by Breidablik, Meland and Lydersen in a cross-sectional study of 16 to 20 years old Norwegian students [51].

### Medication

Medication was measured using two questions. “In the past six months, have you taken antidepressants or sedatives?” with the response alternatives “yes” or “no”. “How often do you self-medicate with non-prescription painkillers?” with response alternatives “never”, “less than a few times each month”, “a few times each month”, “every week”, and “every day”. The response alternatives “every week”, and “every day” were presented as “every week”.

### Anxiety and depression

Anxiety and depression were measured with the Hospital Anxiety and Depression Scale (HADS) developed by Zigmond and Snaith [49,50]. HADS includes two 7-item sub-scales comprising seven questions each for “anxiety” and for “depression” [49]. Each question scores 0–3 points. A total score of 0–7 points indicate “no” anxiety/depression, 8–10 points indicates “possible” mild to moderate symptoms, and 11–21 points indicates a “probable” clinically significant condition of anxiety/depression. HADS has been validated in adolescents with adequate test-retest reliability and factor structure [52] and has a similar sensitivity and specificity as the General Health Questionnaire (GHQ) [53]. Good psychometric properties and a two-factor structure were shown in a Swedish adult population sample [54]. In our calculations of correlations, we used the standardized two-factor structure of “anxiety” and “depression” according to Zigmond and Snaith [49,50] which is the most commonly used [53]. However, other factor structures are recently discussed [55].

### Statistics

Data were analyzed with the Statistical Package for the Social Sciences, version 18, SPSS Inc., Chicago, IL. Differences in proportions were evaluated using the Chi-squared test. Independent sample t-tests were used to compare mean values between groups.

As described above (see Measures), an exploratory factor analysis using principal component analysis with varimax rotation and Kaiser normalisation was conducted for variables on a) subjective health complaints (Table 1), and b) perceived stress including sleeping problems (Table 2). A cut-off point for inclusion was factor loading >0.50, which was the same as used by Lindblad et al. [48]. The chosen cut-off assures that the included items are clearly related to the generated factors. Cronbach’s alpha was used to test reliability and values >0.6 were considered to indicate a sufficient degree of internal consistency.

Correlations were computed using Pearson correlation coefficients. Correlations were computed between the identified factors for subjective health complaints and perceived stress, derived from the factor analysis (Tables 1 and 2), and the standardized factors/sub-scales of HADS-anxiety and HADS-depression from the HADS-instrument [49,50]. A *p*-value ≤0.001 was considered significant.

Since the data has a hierarchal structure consisting of three levels – individual, class and school – a hierarchal mixed model approach was applied to measure the proportion of explained variability at each level. Variability at the individual level refers to individual characteristics, whereas variability at class- or school level refers to conditions shared by all individuals in a certain class or school (e.g. teachers, pedagogy, climate, size of class/school). A variance component, the Intraclass Correlation Coefficient (ICC), was calculated for class level and school level. Dependent variables in the mixed models were the created indexes for pressure and demands, activation/high tempo, sleep problems, psychosomatic problems, headache / upper respiratory infection symptoms, musculoskeletal pain symptoms, anxiety, and depression. One covariate, sex, was used in the models.

## Results

### Socio-demographics

Group socio-demographics are described in Table 3. The majority of respondents were 16 or 17 years old (96.2%), with a mean age of 16.5 years. The majority lived with their mother and father and was born in Sweden. Of the 1027 students participating, 60.7% were girls and 39.3% were boys; 75.8% attended academic programmes and 24.2% vocational study programmes, respectively. In the academic programmes 61.8% were girls and 38.2% were boys, and within vocational programmes 56.3% were girls and 43.7% were boys. The Chi Squared test showed no significant differences between girls and boys regarding socio-demographic characteristics.

**Table 3:** **Socio-demographics of 1027 adolescents in grades 1 and 2 in three public upper secondary schools in northern Sweden (Chi**^**2 **^**test)**

**Socio-demographic variables**	**Total, n (%)**	**Girls, n (%)**	**Boys, n (%)**
***Sex***	1027	623 (60.7)	404(39.3)
***Age***			
16 years	562 (54.8)	335 (59.6)	227 (40.4)
17 years	425 (41.4)	262 (61.6)	163 (38.4)
18 years	39 (3.8)	25 (64.1)	14 (35.9)
*1 missing			
***Study programme***			
Academic programme	774 (75.8)	478 (61.8)	296 (38.2)
Vocational programme	247 (24.2)	139 (56.3)	108 (43.7)
*6 missing			
***Family situation***			
Living with both parents	639 (62.2)	380 (59.5)	259 (40.5)
Living with only one parent	142 (13.8)	87 (61.3)	55 (38.7)
Other family combination	207 (20.2)	130 (62.8)	77 (37.2)
Living alone	39 (3.8)	26 (66.7)	13 (33.3)
***Country of birth***			
Sweden	968 (95.5)	591 (61.1)	377 (38.9)
Other Nordic country	5 (0.5)	3 (60)	2 (40)
Europe or North America	12 (1.2)	7 (58.3)	5 (41.7)
Outside Europe or North America	29 (2.9)	15 (51.7)	14 (48.3)
*13 missing			

No significant differences between boys and girls.

### Subjective health complaints and general self-rated health

Being tired and feeling sad were the most common subjective health complaints (Table 4). Of the girls, 38.8% reported “fairly often/very often” feeling more tired than before compared to 20.3% of the boys (*p* ≤ 0.000). Of the girls 35.0% reported “fairly often/very often” sadness compared to 8.2% of the boys (*p* ≤ 0.000). The most common musculoskeletal problem was neck pain for girls and low back pain for boys. Three-fold more girls (33%) reported headache than boys (11.1%). Significantly more girls than boys reported symptoms of upper respiratory infection. It is noteworthy, that in contrast to the high prevalence of specific health complaints they reported, the majority of the adolescents rated their overall health as “good” (very good/fairly good), with significantly more boys than girls rating their health as “good”. Almost 20% of the girls took over-the-counter pain medicines every week. The use of antidepressants or sedatives was lower, with no significant differences between girls and boys.

**Table 4:** **Prevalence (%) of subjective health complaints expressed as *psychosomatic symptoms, headache and upper infection symptoms *and *musculoskeletal pain symptoms *in the last six months in girls and boys**

**Subjective health complaint indexes**	**Girls (%)**			**Boys (%)**			***P*-value**
	**Never/ seldom**	**Sometimes**	**Fairly often/very often**	**Never/ seldom**	**Sometimes**	**Fairly often/very often**	
***Psychosomatic symptoms variables ª***							
More tired than before	25.1	36.1	38.8	47.7	31.9	20.3	0.000
Sadness	28.9	36.1	35.0	67.8	24.1	8.2	0.000
Acid stomach, stomach ache	60.2	25.0	14.8	86.2	10.9	3.0	0.000
Pain and aches in heart and chest	73.6	16.2	10.1	80.6	13.4	5.9	0.019
Palpitations and extra heart beats	79.9	13.7	6.4	87.6	8.7	3.7	0.000
***Headache and upper respiratory infection symptoms variables ª***							
Headache	34.2	32.6	33.2	61.4	27.5	11.1	0.000
Cold symptoms	38.3	36.5	25.2	58.9	27.7	13.4	0.000
Cough and hoarseness	61.9	26.5	11.6	75.5	18.6	5.9	0.000
Nausea	58.3	26.2	15.6	86.8	10.4	2.7	0.000
***Musculoskeletal pain symptoms variablesª***							
Neck and shoulder pain	40.1	25.4	34.5	67.5	21.4	10.9	0.000
Low back pain	52.3	23.5	24.2	68.2	16.8	15.0	0.000
Pain in extremity joints	65.0	22.3	12.7	71.5	18.6	9.9	0.079
Muscle pain	64.4	25.6	10.0	71.0	20.5	8.4	0.05
**General self-rated health**^**b**^							
Overall good health	79.5			87.8			0.000
**Medication**^**b**^							
Antidepressants or sedatives (yes)	4.7			3.5			0.3
Non-prescription painkillers, every week	17.9			7.2			0.000

Response alternatives were “never/seldom”, “sometimes”, and “fairly often/very often” (Chi^2^ test with p-values for between group differences).

ªSubjective health complaint indexes included variables yielded from factor analysis (see Table 1). ^b^General self-rated health and medication not included in the factor analysis.

### Perceived stress

For all of the stress variables, more girls than boys reported significantly more perceived stress (Table 5). More than half of boys and girls “often/always” perceived demands from school as stressful, and many girls also perceived high inner demands in that way, while few girls and boys perceived demands from home as stressful. Over half (54.7%) of the girls never felt really free, and there were more girls than boys who reported doing many things at the same time. Many girls reported eating rapidly even when they did not have to, and more girls than boys had difficulty in relaxing. Difficulty in falling asleep at night and helplessness were reported more often among girls than boys.

**Table 5:** **Prevalence (%) of perceived stress expressed as *pressure and demands, activation/high tempo *and *sleep problems *during the previous weeks in girls and boys**

**Perceived stress indexes ª**	**Girls (%)**			**Boys (%)**			***P*-value**
	**Never/seldom**	**Sometimes**	**Often/always**	**Never/seldom**	**Sometimes**	**Often/always**	
***Pressure and Demands variables ª***							
I don’t have enough time	28.4	38.3	33.4	39.4	38.4	22.3	0.000
I feel under pressure from school demands	9.8	26.6	63.6	26.1	35.5	38.5	0.000
I feel helpless	58.9	27.1	14.0	79.0	16.3	4.7	0.000
I never feel really free	19.8	25.5	54.7	37.1	27.2	35.6	0.000
I feel under pressure from demands at home	63.0	20.6	16.4	74.2	13.9	11.9	0.003
I feel under pressure from my inner demands	18.3	25.7	56.1	38.9	33.2	28.0	0.000
I don’t feel rested after sleep	20.7	29.7	49.6	34.6	28.0	37.4	0.000
***Activation/High tempo variables ª***							
I rush even if I don’t have to	35.0	33.7	31.3	62.6	26.8	10.7	0.000
I keep a high speed all the day	35.9	39.6	24.5	62.4	23.8	13.9	0.000
I eat rapidly even if I don’t have to	31.7	29.4	39.0	43.1	25.0	32.0	0.006
I do many things at the same time	13.0	34.1	52.8	28.0	40.8	31.2	0.000
I find it difficult to relax	27.1	39.8	33.1	57.3	28.5	14.1	0.000
***Sleep problems variables ª***							
I sleep restlessly and shallow	60.1	25.2	14.6	77.5	16.9	5.7	0.000
I have difficulty falling asleep	40.0	32.1	28.0	54.7	22.3	23	0.000
I wake up early in the morning	72.6	16.1	11.4	83.6	9.4	6.9	0.000
I feel restless	28.4	50.4	21.2	36.7	47.1	16.1	0.035

Response alternatives were “never/seldom”, “sometimes” and “often/always” (Chi^2^ test with p-values for between group differences).

**ª**Perceived stress index included variables yielded from factor analysis. (see Table 2).

### Anxiety and depression

Overall, these adolescents scored higher on the HADS-anxiety scale than on the HADS-depression scale (Table 6). Of the girls, 31.5% scored as “possible” and 27% as “probable” anxiety compared to 21.1% with “possible” and 8.7% with “probable” anxiety among the boys. According to the independent sample t-test, sex/gender differences in signs of depression were small and non-significant. Of the girls, 10% scored as “possible” and 4.1% as “probable” depression compared to 7.9% and 2.5%, respectively, among the boys.

**Table 6:** Prevalence (%) of self-reported anxiety and depression according to the Hospital Anxiety and Depression Scale (mean, SD) in girls and boys (independent sample t-test)

**HADS**	**Girls**	**Boys**	**P-value**
***HADS-anxiety***			0.001*
Total score (mean, SD)	8.5 ± 3.8	5.8 ± 3.3	
*Severity (score range)*			
Normal (1–7 points) (%)	41.5	70.1	
Possible (8–10 points) (%)	31.5	21.1	
Probable (11–21 points) (%)	27.0	8.7	
***HADS-depression***			0.19 (n.s)
Total score (mean, SD)	4.1 ± 3.0	3.5 ± 2.7	
*Severity (score range)*			
Normal (1–7 points) (%)	85.9	89.6	
Possible (8–10 points) (%)	10.0	7.9	
Probable (11–21 points) (%)	4.1	2.5	

*Statistically significant difference at p ≤ 0.001.

### Correlations

Subjective health complaints correlated significantly with all factors of perceived stress, anxiety and depression, and vice versa (see Correlation matrix, Table 7). The strongest correlations were found between the factors “psychosomatic complaints” and “anxiety” (r = 0.71), and between perceived stress expressed as “pressure and demands” and “anxiety” (r = 0.71). Perceived stress in the form of “pressure and demands” was also significantly correlated with “activation/high tempo” (r = 0.68), “psychosomatic problems” (r = 0.63), “depression” (r = 0.52), sleep problems (r = 0.48), “headache/upper respiratory infections” (r = 0.48) and “musculoskeletal pain symptoms” (r = 0.44). “Sleep problems” strongly correlated with “psychosomatic problems” (r = 0.56), “anxiety” (r = 0.53) and stress expressed as “activation/high tempo” (r = 0.50). The psychological factors of “anxiety” and “depression” from HADS were also significantly correlated to each other (r = 0.56).

**Table 7:** Correlations between factors/indexes of perceived stress (pressure and demands; activation/high tempo), sleep, subjective health complaints (headache/upper respiratory infections; musculoskeletal pain), anxiety and depression in adolescents (Pearson correlation coefficients)

**Factors/Indexes**	**Pressure and demands**	**Activation /high tempo**	**Sleep problems**	**Psycho-somatic**	**Headache /upper respiratory infections**	**Musculo- skeletal pain**	**HADS-anxiety**	**HADS-depression**
**Pressure and demands**	1	0.68	0.48	0.63	0.48	0.44	0.71	0.52
**Activation/high tempo**	0.68	1	0.50	0.55	0.44	0.39	0.66	0.39
**Sleep problems**	0.48	0.50	1	0.56	0.41	0.44	0.53	0.39
**Psychosomatic**	0.63	0.55	0.56	1	0.58	0.55	0.71	0.49
**Headache/upper respiratory infections**	0.48	0.44	0.41	0.58	1	0.51	0.52	0.35
**Musculoskeletal pain**	0.44	0.39	0.44	0.55	0.51	1	0.45	0.30
**HADS-anxiety**	0.71	0.66	0.53	0.71	0.52	0.45	1	0.56
**HADS-depression**	0.52	0.39	0.39	0.49	0.35	0.30	0.56	1

All correlations were statistically significant at p ≤ 0.001.

### Hierarchal mixed model analysis

According to the hierarchal mixed model analysis 96–100% of the total variability in the outcome indexes can be explained by individual-level characteristics, such as sex/gender, and not by factors at class- or school-level. The class level and the school level explained 0.6–3.8% (ICC 0.006–0.038) and 0–2.3% (ICC 0–0.023), respectively. Differences between boys and girls remained statistically significant in all outcome indexes even after calculating the proportion of variance at all hierarchal levels.

## Discussion

The main finding in this study among older adolescents is the high prevalence of subjective health complaints and perceived stress, especially among the girls. For nearly all complaints, the proportion of girls reporting “often” or “always” was two to three times higher than among boys. Perceived stress was associated with both physical and psychosomatic symptoms, including sleep problems and psychological symptoms of anxiety and depression. However, the strongest associations were found between perceived stress, psychosomatic symptoms and anxiety. The consistent pattern of gender differences in subjective health and physical and psychological health complaints are in line with findings from several previous national and international studies on young adolescent subjective health [7-10,19,35]. The results of our study indicate that negative trends in subjective health among girls that start in early adolescence seem to persist in late adolescence. It is noteworthy that although there were high levels of subjective health complaints and perceived stress, the majority (83.6%) rated their overall health as “fairly good/very good”. This is similar to a Norwegian study where 88% of the students (16–20 years) rated their health as “good” or “very good” [51].The interpretation of this might be that the symptoms reported, although frequent, are not severe enough to have a significant negative effect on “health”, as characterized by the adolescents. Alternatively, adolescents may interpret “good health” simply as “not being physically sick”. Their definition of health may not include their perceptions of general wellbeing or mental illness. In comparison, Breidablik et al. [51] suggest that adolescents incorporate a broad health definition when describing their health, and that their constructs of self-rated health are associated with several medical, social and psychological background factors. Since self-rated health is seen as an important health indicator, both positive and negative self-rated health during adolescence need to be further studied [51,56,57].

Another finding was that nearly 63.6% of the girls and 38.5% of the boys “often” or “always” perceived as stressful, forms of high pressures and demands from school. The proportion perceiving demands from home in this way was much lower. Thus, the school environment and/or school-work is an important possible stressor and this finding is in line with earlier studies on school-related stress and health [13,24,58]. Perceptions of high self-imposed demands and inner pressure were common, especially among the girls. Similar perceptions of high self-imposed demands and distress were illuminated in a qualitative study comprising young women, aged 16–25 years, who had sought help for stress-related problems [31]. More girls than boys reported helplessness, which can be interpreted as a marker for a low degree of control and perceived inability to handle their situation. This may indicate poor social support. This is consistent with the definition of increased stress as a situation or condition where perceived demands exceed perceived resources and coping abilities [59]. In addition, it reveals lack of individual power and control, which seems to be a key factor relating to girls’ self-esteem and health [58,60].

Also according with our findings, perceived high demands in school and high responsibility-taking have been highlighted as possible stressors and contributors to mental strain in adolescent girls [31,34]. Qualitative studies have illuminated how boys experience fewer demands and less pressure to perform well, and do not report multiple demands as often as girls do [60]. Furthermore, responsibility-taking is interpreted as a power-negotiation strategy for girls in school [60]. Girls seem to take a great deal responsibility for schoolwork as well for other concurrent duties in life [31]. Several studies, point to the importance of balance between demands, control and support for adolescent health and well-being [19,58].

One striking result was the high prevalence of anxiety among both boys and girls, although this was more pronounced among girls. In comparison with a control sample of secondary school girls in a study by Blom et al. [61], the girls in our study had a higher mean value of anxiety. Increased levels of anxiety, as measured by the HAD-scale, may be an aspect of high stress arousal, as this correlates well with the stress indexes of “pressure and demands“ and “activation/high tempo”. Anxiety also correlates with the psychosomatic symptom index consisting of both mental and physical symptoms. This relation between symptoms can be interpreted as a starting-point for stress-related disorders. The rate of depression was lower than that of anxiety. It is possible that the cut-off of eight for possible depression was set too high. White et al. [52] recommend cut-offs of seven (possible mild/moderate depression) and nine (probable clinically significant depression) to capture depression in early stages among adolescents. Reporting feeling tired and sad may be an expression of a depressive mood and warrants attention to the possible development of more severe stress-related disorders and mental ill-health. Aalto-Setälä et al. [62] examined mental health risks in adolescence and found that girls were more likely to be distressed than boys, and that high trait anxiety and somatic symptom scores among adolescents predicted mental distress among women. A longitudinal study [63] found that perceived nervousness and anxiety were strong predictors of premature mortality and psychiatric disorders. Anxiety disorders most typically have their onset in childhood or adolescence [28], and are therefore important to capture in early stages.

Consequently, the strong associations between pressures and demands, activation/high tempo and physical and psychological symptoms must be interpreted as a negative health pattern. Moreover these findings are emphasized by the presence of sleeping problems, as stress impairs sleep. Our results show slightly higher prevalence of sleep-onset difficulties than a study of 15-year olds in Norway [41]. However, that study adressed younger adolescents and may have used a stricter cut-off for sleep-onset difficulties. These signs of sleep-related problems may be related to mental health [64], but also to psycho-physiological strain and theories of allostatic load [65], as these processes may result in chronic deterioration of cardiovascular, immune, gastrointestinal and cognitive systems, in the long run*.* Long-term effects of this type of strain in young age groups are less understood, although relationships between psychosocial stress in childhood and increased stress-responses have been found [66]. Moreover, pain problems and sleeping disturbances starting during childhood and adolescence tend to persist into adulthood [39,40]. In line with the present study, recent studies have addressed severe stress-related disorders of school-burnout [13] and chronic stress [19] among older adolescents.

One key question is why girls report more pressures and demands, stress, and health complaints than boys do. Explanations suggested on both individual and societal levels, include Hyde et al.’s [67] model in which biological, affective, and cognitive vulnerabilities in females interact with negative life-events. Pubertal timing has been investigated in relation to emotional distress in girls [68] and early puberty has been linked to various problem-behaviors [69]. Stattin et al. [69] emphasize the role of contextual mechanisms such as peer-socialization in understanding external problem behavior. Earlier explanatory models, such as the symptom perception theory, emphasize gender differences according to how individuals pay attention to, define and react to symptoms [35,70]; whereas interpersonal stress models focus on girls’ vulnerability to emotional distress connected to social relationships [71].

In contrast, socially-oriented models highlight the importance of contextualizing adolescent distress, including social aspects of performing and constructing gender, and also address unequal power relations [31,34,60]. Girls may develop multiple stress complaints as a result of psychosocial stressors in the environment. The transition to adulthood is a period when adolescent girls seem to encounter socially shaped contradicting expectations and rapidly expanding roles that may be stressful and difficult to manage, particularly if social support is inadequate [31]. Furthermore, Maclean et al. [72] discuss differences in symptom-reporting in relation to gender stereotypes, and conclude that according to norms of masculinity, boys seem reluctant to report health complaints, whereas girls have more complex patterns of symptom-reporting. They also point to similarities, since both boys and girls felt pressured to react to symptoms in a “stoic” way. Hagquist [6] proposes the inclusion of both internalizing and externalizing problems when measuring adolescent mental health, because boys tend to report more externalizing problems and girls more internalizing problems. However, these complex gender-related patterns need to be further addressed. Future studies should pay attention to the balance between gender-related demands, perceived control, and social support, particularly in the school environment, in order to prevent negative strain and stress-related ill-health.

### Limitations and strengths

This study has both a number of limitations, and strengths that are relevant to the interpretation and robustness of the results. Firstly, the cross-sectional design has its given limitations. Since cross-sectional data represent a “snap-shot in time”, our results cannot indicate (one-way directional) causality or symptom development over time. Accordingly, the present results do not explain whether perceived stress caused health complaints, or vice versa. Instead, this study shows significant gender differences in prevalence of perceived stress and interesting (bi-directional) symptom patterns. However, based on results from other studies [24,73,74] and our own clinical experience, we hypothesize that perceived stress and demands may be important explanatory factors in the reported physical/psychological symptoms. Associations between perceived stressors and demands and physical/psychological symptoms are findings of importance to worth further in-depth attention in future studies. Little is still known about long-term consequences of stress and gendered patterns of “school burnout” at young ages [13,19,31].

Another element of uncertainty is how representative the studied group is of a broader population of adolescents, as the sample was limited to three schools in northern Sweden. There was an overrepresentation of girls, particularly in academic programmes; and an underrepresentation of boys, particularly in vocational programmes, as all of the students at the invited schools were not approachable at the time of the survey. The reasons for this were firstly that the study was performed within the constraints of a school setting, and secondly, that we chose to conduct a class-room survey. Teachers had difficulties allocating time for the survey due to a tight schedule, and potential participants at vocational programmes were not approachable because of work placement periods. Furthermore, non-participants could not be reached and analysis of the non-participants was not possible as, for ethical reasons, no record of respondent names was compiled.

However, even given these weaknesses, the participating students represented a relatively large sample of 16–18 years old boys and girls from 49 different classes attending a wide range of educational programmes, both vocational and academic, at the largest public upper secondary schools in the geographical area. A strength of the study that may be connected to the class-room procedure, is its high response rate and low level of missing data. An additional strength is that the questionnaire covered a wide range of physical and mental complaints, including perceived stress, sleep, pain, anxiety and depression – symptoms that are found to be potential predictors for development of prolonged problems or psychiatric problems [39,40,42-44]. The use of an established instrument for measuring anxiety and depression contributed to the value of the knowledge derived.

## Conclusions

In conclusion, this study of older adolescents found significant differences between boys and girls in their subjective health and perceived stress. Both boys and girls experienced stress in the form of high pressure and demands from school which were strongly associated with psychosomatic symptoms and anxiety. The results may indicate a trend toward increased symptoms of severe stress-related disorders, especially among girls. Furthermore, perceived stress, pressure and demands may be important explanatory factors. However, further exploration is needed of how cultural norms and gender-related expectations, including those of the school environment, affect adolescent stress and health.

## Competing interests

No competing interests.

## Authors’ contributions

All authors contributed to the study’s design, the interpretation of data and to the critical revision of the manuscript. MW, EBMO, AÖ and AFW were responsible for contacts with the schools and school-health services, as well as for the data-collection procedure. MW, EBMO and AFW prepared the data, performed the statistical analysis, and drafted the manuscript. All authors read and approved the final manuscript.

## Pre-publication history

The pre-publication history for this paper can be accessed here:

http://www.biomedcentral.com/1471-2458/12/993/prepub
